# Effects of Mathematics Anxiety and Mathematical Metacognition on Word Problem Solving in Children with and without Mathematical Learning Difficulties

**DOI:** 10.1371/journal.pone.0130570

**Published:** 2015-06-19

**Authors:** Yinghui Lai, Xiaoshuang Zhu, Yinghe Chen, Yanjun Li

**Affiliations:** Institute of Developmental Psychology, Beijing Normal University, Beijing, P. R. China; University of Leuven, BELGIUM

## Abstract

Mathematics is one of the most objective, logical, and practical academic disciplines. Yet, in addition to cognitive skills, mathematical problem solving also involves affective factors. In the current study, we first investigated effects of mathematics anxiety (MA) and mathematical metacognition on word problem solving (WPS). We tested 224 children (116 boys, *M* = 10.15 years old, *SD* = 0.56) with the Mathematics Anxiety Scale for Children, the Chinese Revised-edition Questionnaire of Pupil’s Metacognitive Ability in Mathematics, and WPS tasks. The results indicated that mathematical metacognition mediated the effect of MA on WPS after controlling for IQ. Second, we divided the children into four mathematics achievement groups including high achieving (HA), typical achieving (TA), low achieving (LA), and mathematical learning difficulty (MLD). Because mathematical metacognition and MA predicted mathematics achievement, we compared group differences in metacognition and MA with IQ partialled out. The results showed that children with MLD scored lower in self-image and higher in learning mathematics anxiety (LMA) than the TA and HA children, but not in mathematical evaluation anxiety (MEA). MLD children’s LMA was also higher than that of their LA counterparts. These results provide insight into factors that may mediate poor WPS performance which emerges under pressure in mathematics. These results also suggest that the anxiety during learning mathematics should be taken into account in mathematical learning difficulty interventions.

## Introduction

Problem solving is “cognitive processing directed at achieving a goal when no solution method is obvious to the problem solver” (p. 287) [1]. As an important component of mathematical problem solving, Word problem solving (WPS) “involves knowledge about semantic construction and mathematical relations as well as knowledge of basic numerical skills and strategies” (p. 1) [2]. For example, a word problem often presents a story (e.g. “Xiaoming bought five pencils, and Xiaohong took three of them. How many pencils does Xiaoming have now?”). Learning how to solve word problems has long been difficult for students and has gained attention in the field of mathematical development [3].

Mathematical problem solving is shaped by affective and cognitive factors [4 – 6]. Mathematics anxiety (MA) was one of the first affective features which were systematically investigated in the mathematics learning domain [7]. Richardson and Suinn (p. 551) [8] define MA as “involving feelings of tension and anxiety that interfere with the manipulating of numbers and the solving of mathematical problems in a wide variety of ordinary life and academic situations.” The Mathematics Anxiety Rating Scale (MARS) measures MA in adults and is popular among educators [9]. The Mathematics Anxiety Scale for Children (MASC) [10] is developed based on the MARS, and used to measure MA in children. MA is an important factor that impedes one’s mathematical problem solving success [11]. High levels of anxiety were found to be related to less efficient mathematical problem solving [6].

In recent years, researchers have made concerted efforts to identify and understand the cognitive mechanisms that underlie children’s word problem solving. Such mechanisms include working memory, processing speed, executive functioning, etc. (e.g. [12–14]) Among various cognitive resources which have been theoretically and empirically investigated in relation to WPS, early research findings have highlighted that metacognition develops alongside general cognitive ability and might be even more effective than general aptitude in predicting mathematics performance [15–17].

The classical concept of metacognition consists of three primary components, i.e. metacognitive knowledge, metacognitive experience, and metacognitive skills [18]. Here, we used Panaoura and Philippou’s concepts [19]. These concepts were consistent with the scale that used to measure mathematical metacognition in the present study. They [19, 20] considered metacognition as mainly indicative of awareness (e.g. self-image) and the monitoring of one’s own cognitive system and its functioning (e.g. self-regulation). As a component of metacognitive knowledge, self-image concerns personal strengths and limitations relative to the abilities of others. Self-image-related terms include self-consciousness and self-evaluation (e.g. pupils’ beliefs about, and self-efficacy with respect to, their abilities) [19, 20]. Characterized by the processes of coordinating and steering cognition, self-regulation reflects the ability to strategically use cognitive knowledge to achieve cognitive goals, particularly when cognitive obstacles need to be overcome [19, 21]. They also suggested that strategies and motivation are two more dimensions of metacognitive ability, although self-image and self-regulation had a relatively strong relationship with metacognitive performance [19]. Strategies concerned the approaches pupils used in order to monitor the problem solving process. Using strategies is an important metacognitive skill. Finally, motivation refers to eliciting pupils’ beliefs about their efforts, about their will on their performance, and about the impact of their parents and teachers. Motivation is an important energizing factor of metacognition and can activate the self-regulation process.

A number of studies (e.g. [22–24]) have explored the effect of metacognition on mathematical problem solving. The meta-skills of children in grades 3 and 4 are strongly related to their numerical and geometrical problem solving abilities [22], and metacognitive ability also predicts performance in a WPS task [21, 24]. Moreover, metacognition can be trained to improve WPS ability [25, 26].

In summary, metacognition and MA are important cognitive and affective variables that are related to students’ mathematical performances and mathematical problem solving. What is not so clear is how MA and metacognition to be related to mathematics problem solving performance. Some research supports the conclusion that MA may impede mathematics performance by affecting cognitive process [27–29], but only a few studies have explored the relationship between MA and metacognition in learning: Students who experienced lower anxiety used more metacognitive regulation [30, 31]. Children with positive beliefs about social support experienced much less math anxiety than those who did not [32]. The attribution of failure or success may also be correlated to test anxiety [33].

Additional research examined the influences of test anxiety and metacognitive word knowledge on reading comprehension performance [34]. This study found that test anxiety exerts a negative influence on students' metacognitive performances [34]. Although that experiment focused on the reading domain, its results suggest that metacognition and anxiety are related to performance in other learning domains. Recently, Legg and Locker [35] measured metacognitive awareness and mathematics anxiety in adults. They hypothesized that individuals with high metacognition and high mathematics anxiety would tend to display poorer mathematics performance. However, the results showed that at high anxiety levels, individuals performed increasingly worse as their metacognition scores decreased, but the performance did not differ at low anxiety levels regardless of the level of metacognition. However, this study did not investigate the holistic relationship between MA, mathematical metacognition, and mathematical problem solving in children.

The relationship between MA, metacognition, and mathematical performance may be multi-directional. For example, MA may lead to poor mathematical performance and vice versa in the longitudinal view. For example, Ma and Xu [36] used longitudinal panel analysis throughout junior and senior high school. They found prior mathematics achievement to be negatively related to later mathematics anxiety. Jansen et al. [37] used a computer-adaptive program that adjusted the difficulty of each problem to the individual’s ability level to manipulate children’s experience of success in mathematics. They did not find that experiencing mathematical success affected the level of mathematics anxiety.

In the current study, our purpose was to determine whether (a) mathematics anxiety was negatively related to word problem solving; (b) metacognition could counter this negative relation; or (c) a potential compensatory relationship between metacognition and mathematics anxiety on WPS might exist in children. We tested the path model of “MA-> metacognition-> WPS”, and predicted that metacognition would mediate the relationship between MA and children’s WPS performance.

This path model was to some extent inspired by Kulm’s model for attitude-behavior relationships [38]. Kulm developed the model as a source of hypotheses for research on attitudes toward mathematics. Hypotheses generated from the model have a general form: “Hypothesis: Given attitude factor A (+ or-), mediating factor B (+ or-), and learning situation C (+ or-), the subject's response will be (positive or negative)” (p. 380) [39]. Although attitude is not the same as emotional factors, and although aspects of the learning situation, such as children’s perception of the importance of the task were not measured, this model inspired the current investigation of the relationship among negative attitudes and emotion (anxiety about mathematics), mediating factors such as mathematical metacognition, and specific behavioral responses (word problem solving performance).

After investigating the relationship between MA, mathematical metacognition, and WPS, we examined MA and metacognitions of the children at four mathematical learning achievement levels, i.e. high achieving (HA), typical achieving (TA), low achieving (LA), and exhibiting mathematical learning difficulty (MLD).

Mathematical learning difficulty refers to a specific learning deficit that affects the normal acquisition of mathematical skills [40], and a preponderance of researchers have relied on standardized achievement tests often in combination with measures of intelligence (IQ), to identify MLD [41]. Although the criteria for identifying children with MLD remain unresolved, researchers commonly use cutoff scores on standardized achievement tests for grouping [42, 43]. The current study used standardized mathematics achievement scores to define the four mathematics achievement groups.

A number of studies have shown that MLD children exhibit poorer WPS abilities than do their typical peers [23, 44, 45], and that they are typically poor mathematical problem solvers with restricted cognitive and metacognitive knowledge [17, 46, 47]. MLD children tend to overestimate their mathematics abilities [48, 49], to respond impulsively, to fail to verify or evaluate answers, and to settle for the first answer in mathematics tasks [45]. Moreover, these children use fewer metacognitive strategies and exhibit more nonproductive behaviors than do high achievers, when solving mathematics problems [17, 47]. Desoete, Roeyers, and Buysse [50] argued that above-average mathematical problem solvers did better on metacognitive knowledge (declarative, procedural, and conditional knowledge), skill (prediction, planning, monitoring, and evaluation skills), and attribution to effort than average performers, yet only prediction and evaluation skills can differentiate children with MLD from their average performing peers. Prediction skill was measured by asking children to look at exercises without solving them and to predict whether they would be successful in this task, and evaluation skill refers to self-judging the answers and to the process of arriving at these answers. In some reports [50, 51], the majority of children with MLD in Grade 3 made inaccurate predictions and exhibited evaluation skills insufficient for word problems that involve language-related and mental representation tasks.

Furthermore, Rosenzweig et al. [17] found that the students with learning difficulties (LD) had significantly more nonproductive metacognitive verbalizations than both the low and average achievers on difficult problems. This suggests that students with LD might not have the metacognitive resources (ability to self-monitor, self-instruct, self-question, and self-correct statements/questions directly related to solving the problem) available to apply to the tasks that their low achieving peers have, when confronted with problems that are difficult or that they perceive to be difficult.

In the current study, we distinguished LA from MLD for the following reasons. In some studies, the children in the lowest 25% (the highest cutoff criterion was the 46^th^ percentile) were placed in the MLD group [44, 52]. Some researchers [53] believe that studies with high cutoffs may actually measure causes of low math achievement rather than causes of MLD. In the longitudinal view, the growth rate of mathematical and math-related skills in these two groups may differ [43]. Although the current study did not investigate the rate of development of mathematical ability in the two groups, the existence of any difference between the LA group (children who typically scored between the 11^th^ and 25^th^ percentiles on mathematical achievement performance) and the MLD group (children who scored at or below the 10^th^ percentile) related to metacognitive and affective features. We also hoped that these data may offer information on the selection of children for special education or related interventions.

Additional studies have examined the relationship between anxiety and learning difficulty. A meta-analysis of 58 empirical studies on school-aged students revealed that the learning disabled individuals suffered more trait anxiety (defined as general anxiety that is stable over time and across settings) than did their typical peers and that their level of test anxiety was significantly related to reading and mathematics achievement scores [54, 55]. According to the definition of mathematical anxiety, MA may both reflect the anxiety aroused in an assumed test situation and the anxiety of children’s ordinary life related to mathematics. Early research [38] suggested that mathematics anxiety may be positively related to test anxiety and this correlation seemed to be stronger than that of mathematical anxiety and trait anxiety. Recently, Wu et al. [56] conducted a study in which they did not find any relationship between mathematics anxiety and trait anxiety in second and third graders.

Test anxiety should be measured in certain test situations, and the level of test anxiety may vary with the interval between the time of the test anxiety measurement and the tests. Trait anxiety may be harder to mediate over the short term via metacognition on word problem solving than the other two types of anxiety. Considering these reasons, we focused on mathematical anxiety. Because MLD is likely to show some of the same characteristics as other forms of LD, we therefore expected that the MLD children would experience higher levels of MA and lower levels of mathematical metacognition compared to their typical peers.

Accordingly, the goal of the present research was to answer two specific research questions. First, we investigated the mediating role of mathematical metacognition between the relationship of mathematics anxiety and word problem solving. Second, we divided the children into four mathematical achievement groups, and investigated group differences in mathematical metacognition and mathematics anxiety.

## Methods

### Ethics Statement

This research was approved by the local ethical committee of Beijing Normal University. We obtained informed written consent from the next of kin, caretakers, or guardians on behalf of the minors/children participants involved in the study according to the institutional guidelines of Beijing Normal University.

### Participants

We tested 224 10-year-old Chinese children (116 boys, *M* = 10.15 years old, *SD* = 0.56) in the fourth grade from three elementary schools. All of the children were of medium socioeconomic status, and their monthly family incomes near or slightly above national averages. We used this sample to test the mediating effect of metacognition. Because the definition of MLD emphasized children of normal intelligence [57], we excluded 7 children of extremely low non-verbal intelligence score (see below), leaving a sample of 217 for grouping.

The present study focused on the children who met the following criteria across two successive semesters. We considered a child’s standardized mathematical achievement scores consistent, if those scores fell within the same range (specified below) over one year and fell within the 95% confidence intervals for that range throughout the year. We used the consistent mathematical achievement scores across the year for grouping for the following reasons. Some studies [52] used a single mathematical achievement score to identify the children with MLD, but some others [58] suggested that these criteria may lead to false positives. In these cases, children will be classified as MLD who in fact typically show improved achievement scores in later grades. More recently, while some studies [59] still use one mathematical achievement score to identify children with MLD, others [60] have begun to use longitudinal analysis to collect children’s mathematical achievement scores for two or more years. If children consistently fell into the same range, they were classified into the same group. In this way, although the present study was not a longitudinal study, in order to reduce the possible biases that one mathematical achievement score might have, we used two mathematical achievement scores to classify children.

We used cut-off scores on standardized mathematics achievement tests as a proxy classification. Eighteen children (12 boys) met the criteria for MLD due to mathematical achievement scores that fell at or below the 10^th^ percentile. We selected this percentile to align with the reported prevalence of MLD (~6–11%) [61, 62], and this same cut-off point has been used in many previous investigations [51, 61, 62]. Further, 28 children (11 boys) met the low achieving (LA) criteria, with scores between the 11^th^ and 25^th^ percentiles. This range for LA children was used in some research (e.g. [63]). Although the 25^th^ percentile was used in some earlier research as the criterion for MLD [52], that value is inconsistent with reported MLD prevalence and may obscure underlying differences. Additionally, 151 children (78 boys) met typically achieving (TA) criteria, with scores between the 25^th^ and 95^th^ percentiles, and 18 children (10 boys) met the high achieving (HA) criteria, with scores above the 95^th^ percentile. The 95^th^ percentile was selected for subgrouping HA children because it is a commonly used criterion for school placement in gifted and talented programs, and has been used in earlier studies of HA students [64, 65].

### Materials and procedure

In January, we first measured children’s verbal intelligence individually. Then, two days later, we assessed children’s non-verbal intelligence in six different classes from three primary schools (the number of children in each class ranged from 33 to 47). We also recorded the pupils’ first final mathematics test performances. One month after the first final mathematics test, their metacognitive abilities in mathematics and their MA scores were recorded. One day later, we administered the WPS test. This time interval may help reduce the possible influence of math achievement task on children’s mathematical metacognition and MA scores. Finally, we collected the second final mathematics test scores in July. All tests except the verbal intelligence test were administered collectively in children’s classrooms. Materials were all presented in Chinese and that quotes from them in this paper are translations.

#### Intelligence measures

The present study utilized the Chinese revised edition of Raven’s Standard Progressive Matrices (RPM-CR) [66] to measure the participants’ non-verbal intelligence. We administered the verbal comprehension subtests of the Wechsler Intelligence Scale for Children-Fourth Edition (WISC-IV; Chinese Version) [67] to screen the children’s verbal intelligence. The verbal and non-verbal intelligence scores were significantly correlated (*r* = .22, *p* < .01). We excluded 7 children (5 boys, *M* = 10.19 years old, *SD* = 0.74) from the initial pool when grouping children into different mathematical achievement levels, because their Raven's matrices scores were in the bottom 5% based on Chinese National Raven’s age-appropriate norms. This exclusion was based on the definition of MLD, which emphasized that those children have normal intelligence [57]. In addition, we compared both verbal and non-verbal IQ in the four achievement groups using multivariate analysis of variance (Manova). Group differences were significant for both verbal and non-verbal IQ, *F* (3, 213) = 72.42, *p* < .001, ƞ_p_^2^ = .51, 1 - β = .98; *F* (3, 213) = 2.86, *p* < .05, ƞ_p_^2^ = .04, 1 - β = .68. Because the results showed that these groups were not equivalent in general intelligence, it was necessary to control for IQ score.

#### Mathematical achievement measures

We used the scores from the final mathematics examinations over the two previous semesters to evaluate the students’ mathematical achievement. These two tests were developed by the Education Committee of the Haidian District of Beijing, and followed the Chinese mathematics curriculum standards for full-time compulsory education [68]. Total scores could range from 0 to 100 and examined numerical abilities (30 items, 60 points), visual-spatial abilities (10 items, 10 points) and mathematics application abilities (5 items, 30 points). The internal consistency reliabilities were high (Cronbach’s α = .90, .92, and .88; respectively). The correlation of numerical abilities and visual-spatial abilities was non-significant (*r* = .11, *p* = .13), the correlation of numerical ability and mathematics application abilities was significant (*r* = .24, *p* < .01), and the correlation of visual-spatial abilities and mathematical application abilities was also significant (*r* = .47, *p* < .01). Because the visual-spatial abilities did not reflect the pure mathematical ability, we removed these scores before analysis and used the mean score of the two mathematical achievement scores in the data analyses (see Results). Both of the tests were conducted in the schools and administered by two teachers and one experimenter.

#### Mathematical metacognition

We assessed mathematical metacognition using the Chinese revised-edition Questionnaire of Pupil’s Metacognitive Ability in Mathematics, which was developed by Panaoura and Philippou [19] and revised by Hao et al. [69] This questionnaire contains 30 5-point Likert-type items (1 = never, 5 = always). Items evaluate the following four factors: Self-Image (Cronbach’s α = .81), 7 items that examine the pupils’ beliefs and self-efficacies about their abilities (e.g. “I know how to remember the knowledge of mathematics that I have learned”); Self-Regulation in Mathematics (α = .82), 7 items that examine the pupils’ abilities to clarify the targets of problems, understand mathematical concepts, apply knowledge to generate solution strategies and monitor their progress toward solutions (e.g. “To solve the math problem, I'll try a variety of methods and then determine the final method”); Strategies (α = .90), 12 items that examine the strategies that the pupils use to solve problems and overcome cognitive obstacles (e.g. “I'll draw pictures to help myself to better understand difficult mathematical questions”); and Motivations (α = .68), 4 items that elicit the pupils’ beliefs about the effects of their efforts and those of their parents and teachers on their performances (e.g. “Parents believe that I can learn math well”). Participants rate themselves with respect to each of the statements. The ratings for the items which made up each factor were averaged to give factor scores used in the statistical analyses. A confirmatory factor analysis (CFA) for the present data set indicated a good fit for a four-factor solution (*χ* ^2^ = 711.25, *df* = 399, *χ* ^2^/*df* = 1.78, *RMSEA* = 0.07, *CFI* = 0.97).

#### Mathematics anxiety

We revised the Mathematics Anxiety Scale for Children (MASC) [10]. This scale contains 22 4-point Likert-type items (1 = never anxious, 4 = very anxious). An exploratory factor analysis on our revision yielded two factors consistent with Plake and Parker [70]. The first factor was labeled *Learning Mathematics Anxiety* (*LMA*). It was related to activities during learning or in the process of studying mathematics (e.g. watching a teacher solve an algebraic equation on the blackboard or listening to a lecture in a mathematics class). Cronbach’s α for this factor was .84. The second factor was labeled *Mathematics Evaluation Anxiety* (*MEA*). It was related to evaluations of mathematic or statistical learning (e.g. being given a surprise quiz in a mathematics class). Cronbach’s α for the second factor was .89. The ratings for items making up each factor were averaged to allow comparison. A confirmatory factor analysis indicated a good fit for a two-factor solution (*χ* ^2^ = 413.01, *df* = 208, *χ* ^2^/*df* = 2.04, *RMSEA* = 0.07, *CFI* = 0.94).

#### Word problems

**Fig 1:**
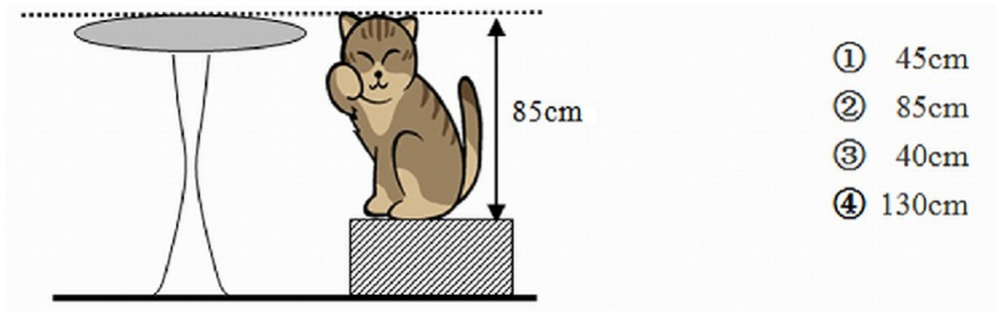
Sample WPS tasks: *Kitty likes resting on the bench*, *and the bench is 45 cm high*. How high is the table?

The children attempted to solve 10 word problems that required them to incorporate their knowledge of mathematics involved in scenarios that would have been familiar from their daily lives, and that depend on their knowledge about magnitude relationships. The word problem measures in the present study are a part of a standardized battery that measures children’s mathematical abilities [71]. The problems included addition, subtraction, multiplication and division (for the full set of problems, Cronbach’s *α* = .69), and all the problems were similar in size (e.g. 2-digit + 2-digit addition, etc.) An example of these questions is shown in Fig 1. Three elementary mathematics teachers who each had extensive teaching experience evaluated the difficulties of the problems based on a 5-point Likert scale (1 = quite easy, 5 = quite hard) and agreed that the average degree of difficulty was moderate (*M* = 2.67, *SD* = 0.24). The Kendall coefficient of concordance (*W*) among the teachers was .93. Participants were asked to compute each problem. Each participant was given two pieces of scratch paper, and they had free access to scratch paper. If the answer was correct, it earned one score point; otherwise, no point was awarded. Scores, therefore, could range from 0 (no problems solved correctly) to 10 (all problems solved correctly). The students had 45 minutes to solve the problems.

## Results

### Effects of MA and mathematical metacognition on WPS

We applied structural equation modeling using Mplus 7.0 to examine the hypothesized models. In the models, mathematical metacognition partially mediated the effect of MA on WPS after controlling for IQ. Because the correlation between the verbal IQ score and WPS was not significant (*r* = .12, *p* = .07), we only controlled for the non-verbal IQ score (*r* = .41, *p* < .01).

We first evaluated the measurement model to assess whether latent variables were well represented by indicator variables. The confirmatory factor analysis was conducted with four latent factors and eight observed variables. The latent variable Metacognition was indexed by 4 indicators (Self-Image, Self-Regulation, Strategies, and Motivation). The latent variable MA was indexed by 2 indicators (Learning mathematics anxiety and Mathematics evaluation anxiety). WPS and non-verbal IQ were each represented by a single indicator with the error variance fixed to zero. The estimation of the measurement model revealed a satisfactory fit to the data: *χ* ^*2*^ = 36.18, *df* = 16, *χ* ^*2*^ */df* = 2.26, *RMSEA* = 0.075, *TLI* = 0.96, *SRMR* = 0.039, *CFI* = 0.98. All the factor loadings for the indicators on the latent variables were significant (*ps* < .001) and the standardized factor loadings ranged from 0.70 to 0.95, indicating that all the latent factors were well represented by their respective indicators.

**Fig 2:**
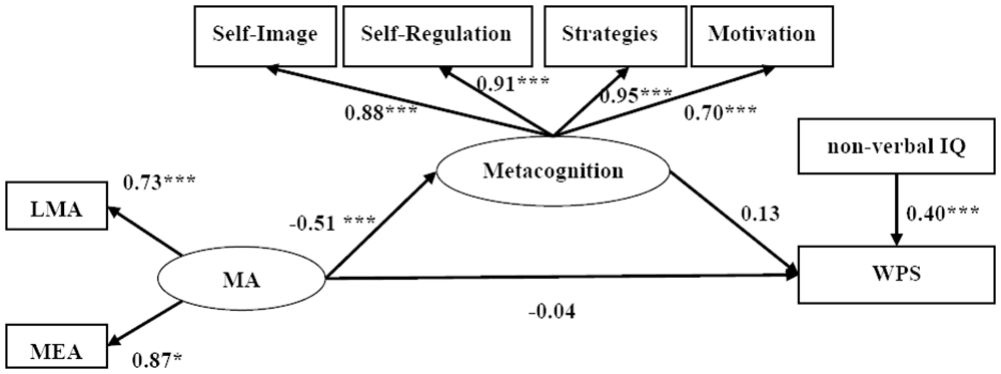
Partially mediated structural equation modeling of mathematics anxiety, mathematical metacognition, and word problem solving with IQ partialled out. MA = mathematics anxiety, LMA = learning mathematics anxiety, MEA = mathematical evaluation anxiety, WPS = word problem solving. * *p* < .05. ** *p* < .01. *** *p* < .001.

To test meant to assess the mediating role of mathematical metacognition between MA and WPS, we constructed a partially mediated model (Model 1) for all 224 participants (the partial correlation matrix is shown in Table 1). In this model, mathematical metacognition partially mediated the effect of MA on WPS after controlling for the non-verbal IQ (see Fig 2).

**Table 1:** Summary of the partial correlations, means, and standard deviations for scores on mathematical metacognition, mathematics anxiety, and word problem solving, after controlling for the Raven’s Standard Progressive Matrices scores and verbal comprehension subtest scores.

	1	2	3	4	5	6	7	8
**1. Self-image**	1							
**2. Self-regulation**	.80**	1						
**3. Strategies**	.83**	.86**	1					
**4. Motivation**	.62**	.63**	.67**	1				
**5. LMA**	-.34**	-.33**	-.34**	-.28**	1			
**6. MEA**	-.37**	-.39**	-.44**	-.31**	.65**	1		
**7. WPS**	.18**	.14*	.14*	.18**	-.19**	-.06	1	
**8. Mathematics score** ^**a**^	.28**	.21**	.19*	.16	-.24**	-.11	.28**	1
***M***	3.71	3.67	3.70	3.87	1.51	2.11	5.93	81.63
***SD***	.84	.84	.82	.89	.44	.65	2.37	12.93

Self-Image, Self-Regulation, Strategies, and Motivation were four dimensions of the mathematical metacognition. LMA = learning mathematics anxiety, MEA = mathematical evaluation anxiety, WPS = word problem solving.

*p < .05.

**p < .01.

***p< .001.

^a^. The mathematics score was the mean score of the two final mathematics achievement tests with the visual-spatial abilities question scores excluded.

**Fig 3:**
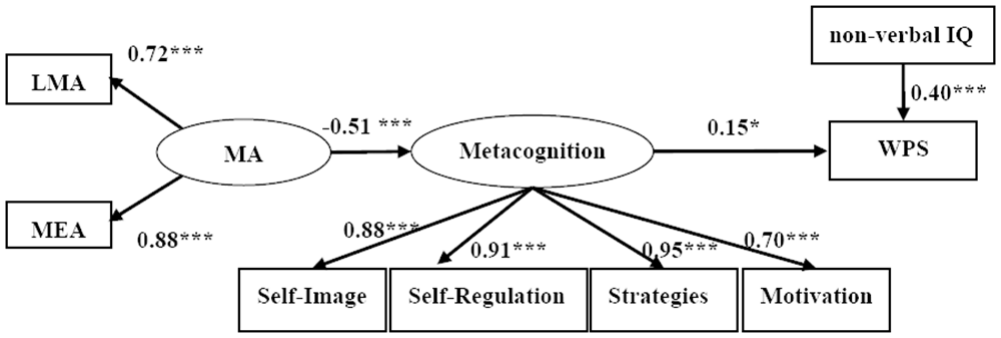
Fully mediated structural equation modeling of mathematics anxiety, mathematical metacognition, and word problem solving with IQ partialled out. MA = mathematics anxiety, LMA = learning mathematics anxiety, MEA = mathematical evaluation anxiety, WPS = word problem solving. * *p* < .05. ** *p* < .01. *** *p* < .001.

Model 1 revealed a good fit to the data (*χ* ^*2*^ = 37.54, *df* = 18, *χ* ^*2*^ */df* = 2.08, *RMSEA* = 0.07, *TLI* = 0.97, *SRMR* = 0.05, *CFI* = 0.98). However, the standardized path coefficient from mathematics anxiety (MA) to word problem solving (WPS) was non-significant (*ß* = -0.04, *p* = .71), as was the path from Metacognition to word problem solving (*ß* = 0.13, *p* = .12). Consequently, a fully mediated model (Model 2) was tested (see Fig 3), which also exhibited a good fit to the data (*χ* ^2^ = 37.72, *df* = 19, *χ* ^2^/*df* = 1.99, *RMSEA* = 0.07, *TLI* = 0.97, *SRMR* = 0.05, *CFI* = 0.98). No significant Chi-square difference existed between Model 1 and Model 2, Δ*χ* ^2^ = 0.18, Δ*df* = 1, Δ*χ* ^2^/Δ*df* = 0.18, *p* > .05. Because there was no significant difference between the models according to the fit indices, the parsimony of Model 2 suggested that its fit was more satisfactory.

The results of Model 2 revealed a significant negative path from the latent MA variable to the latent variable Metacognition (*ß* = -0.51, *p* = .001) and a significant positive path from Metacognition to WPS (*ß* = 0.15, *p* = .02).

We generated 1,000 bootstrapping samples from the original data via random sampling. The indirect effect of metacognition from MA to WPS was -0.08, and the associated 95% confidence intervals were -0.14 to -0.012. The intervals did not overlap with zero; thus, Metacognition exerted a significant indirect effect on WPS via MA.

We also ran both the partially mediated model and fully mediated model with MA as a mediator, and these results are given in the Supporting Information (S1 Table, S1 Fig and S2 Fig). The Supporting Information shows that, in model 3 (the partially mediated model), the paths from the latent MA variable and Metacognition variable to WPS were non-significant. In model 4 (the fully mediated model), the path from MA to WPS was also non-significant. MA was not found to have any mediating effect.

### Group differences in mathematical metacognition and MA

First, we used Hierarchical Regression Analyses to explore the prediction of mathematics achievement scores from mathematical metacognition and MA, when the effect of IQ was partialled out (see Table 2). For the purity of the mathematics measure, the visual-spatial question scores were excluded. All the scores in the regression were standardized. Based on the fact that the prediction was significant, we employed analysis of covariance (ANCOVA) to compare the differences related to mathematical metacognition and MA among the MLD, LA, TA, and HA groups. In order to avoid bias due to the grossly larger number of TA participants, while maintaining a sample size adequate to insure statistical stability, we randomly selected 30 individuals from the TA group. We did this using the Rand (random number) function of the 2010 edition of Microsoft Excel to randomly select 30 subjects (14 boys, *M*_age_ = 10.22 years) from the 151 participants in the TA group. Table 3 displays the means and standard deviations of the variable measures for the four groups.

**Table 2:** Hierarchical Regression Analysis predicting mathematics achievement scores^a^ from the mathematical metacognition and mathematics anxiety with the effect of IQ partialled out.

		*R* ^2^	Δ*R* ^2^	*F*	β	*T*
**Step 1**	IQ	0.47		95.44***		
**Step 2**		0.51	0.04***	35.99***		
	Self-image				0.20	2.27*
	Self-regulation				0.11	1.11
	Strategies				-0.11	-1.00
	Motivation				-0.03	-0.51
**Step 1**	IQ	0.47		95.44***		
**Step 2**		0.50	0.03**	52.10***		
	LMA				-2.51*	-2.95**
	MEA				0.13	

Self-Image, Self-Regulation, Strategies, and Motivation were four dimensions of the mathematical metacognition. LMA = learning mathematics anxiety, MEA = mathematical evaluation anxiety.

*p < .05.

**p < .01.

***p < .001.

^a^. The mathematics achievement score was the mean score of the two final mathematics achievement tests with the visual-spatial abilities question scores excluded.

**Table 3:** Means and standard deviations for four mathematics achievement groups on measures of mathematical metacognition, mathematics anxiety and word problem solving.

	MLD (n = 18)	LA (n = 29)	TA (n = 151)	TA sub-sample (n = 30)	HA (n = 19)
	*M* (*SD*)	*M* (*SD*)	*M* (*SD*)	*M* (*SD*)	*M* (*SD*)
**Self-Image**	3.21 (0.79)	3.54 (0.80)	3.69 (0.87)	3.76 (0.84)	3.98 (0.81)
**Self-Regulation**	3.25 (0.76)	3.52 (0.79)	3.66 (0.85)	3.84 (0.72)	4.03 (0.85)
**Strategies**	3.50 (0.82)	3.57 (0.88)	3.66 (0.82)	3.84 (0.71)	3.94 (0.89)
**Motivation**	3.75 (0.94)	3.59 (1.01)	3.86 (0.86)	4.07 (0.64)	4.09 (1.04)
**LMA**	1.97 (0.53)	1.66 (0.39)	1.57 (0.44)	1.55 (0.42)	1.54 (0.51)
**MEA**	2.21 (0.56)	2.23 (0.72)	2.06 (0.62)	2.16 (0.74)	2.03 (0.64)
**WPS**	4.28 (2.14)	6.83 (1.89)	6.01 (2.29)	5.97 (3.11)	6.47 (2.39)

Note. LMA = learning mathematics anxiety, MEA = mathematical evaluation anxiety. WPS = word problem solving. MLD = mathematical learning difficulty, LA = low achieving, TA = typical achieving, HA = high achieving. Self-Image, Self-Regulation, Strategies, and Motivation were four dimensions of the mathematical metacognition. Mean comparisons of all the statistics above between these two TA groups were similar; *F* values ranged from 0.01 to 1.56, and the probability values ranged from .21 to .92.

#### Group differences in mathematical metacognition

**Fig 4:**
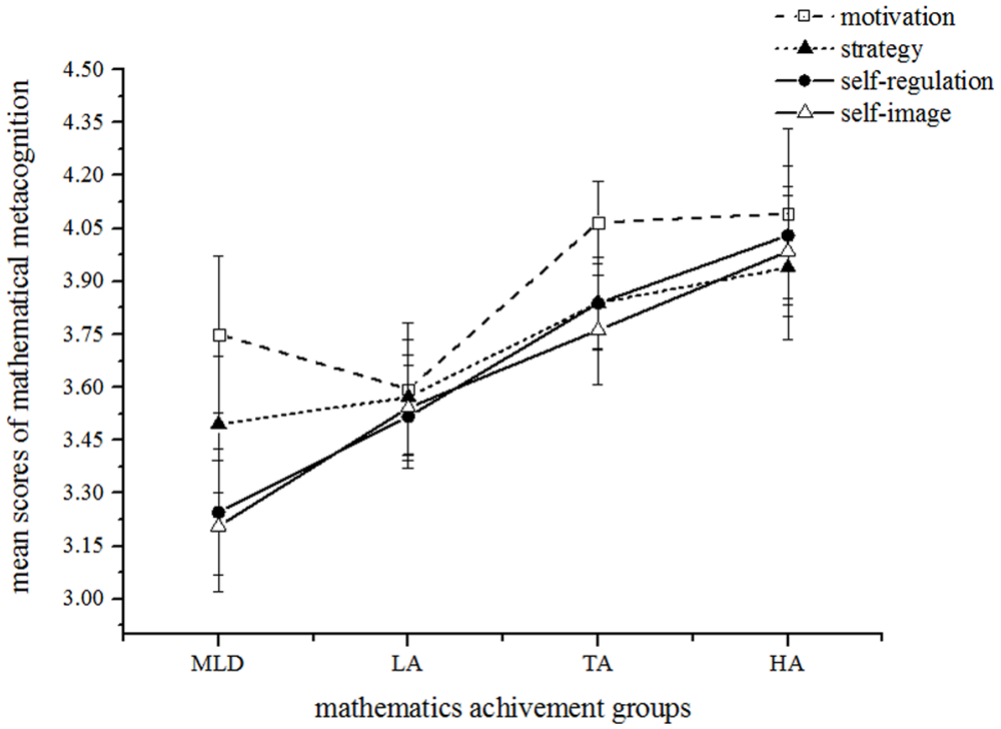
Mean scores for all dimensions of the mathematical metacognition of four mathematics achievement groups. MLD = mathematical learning difficulty, LA = low achieving, TA = typical achieving, HA = high achieving. LMA = learning mathematics anxiety, MEA = mathematical evaluation anxiety.

The regression analysis above (Table 2) showed that Self-image significantly predicted mathematics achievement. Based on these results, we conducted an ANCOVA, using the mathematics achievement groups as the independent variable and the self-image scores as the dependent variable. When controlling for IQ, the main effect of mathematics achievement group was significant, *F* (3, 211) = 3.84, *p* < .05, ƞ_p_^2^ = .11, 1 - β = .81. Post hoc comparisons using the least significant differences (LSD) procedure with an alpha value of .05 revealed that the self-image in children with MLD (*M* = 3.21) was significantly lower than TA and HA groups (*M*_TA_ = 3.76, *p* = .005; *M*_HA_ = 3.98, *p* = .001). The difference between the MLD and LA groups was approaching significance (*M*_LA_ = 3.54, *p* = .05). Fig 4 shows these outcomes. We also used the entire TA group (*n* = 151) to run the ANCOVA, and the results showed that the group differences in self-image were significant, *F*(3, 211) = 3.77, *p* < .05, ƞ_p_^2^ = .05, 1 - β = .81.

#### Group differences in Mathematics Anxiety (MA)

**Fig 5:**
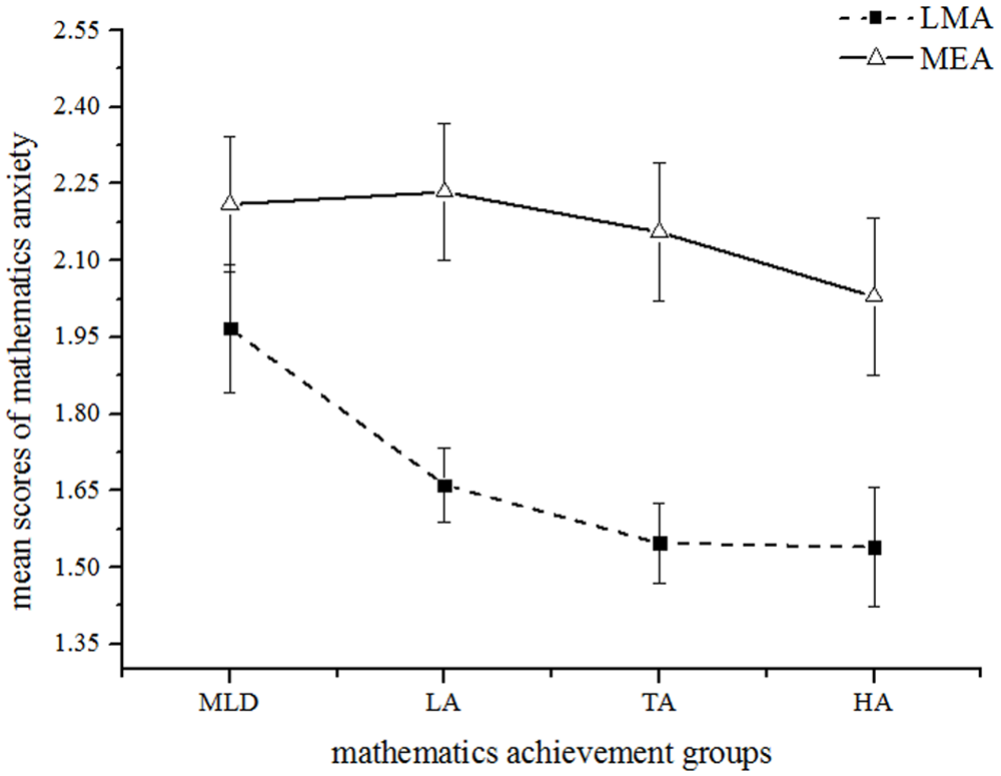
Mean scores for the dimensionalities of MA of four mathematics achievement groups. MLD = mathematical learning difficulty, LA = low achieving, TA = typical achieving, HA = high achieving. LMA = learning mathematics anxiety, MEA = mathematical evaluation anxiety.

The regression analysis above (Table 2) also showed that learning mathematics anxiety (LMA) can significantly predict mathematics achievement. So we conducted an ANCOVA using the mathematics achievement groups as the independent variable and LMA score as the dependent variables, IQ as a covariate. The main effect of group was approaching significance, *F*(3,90) = 2.67, *p* = .05, ƞ_p_^2^ = .08, 1 - β = .63. On average, the MLD group (*M* = 1.97) showed significantly higher LMA scores than did the LA group (*M* = 1.66, *p* = .018), the TA group (*M* = 1.55, *p* = .009), and the HA groups (*M* = 1.54, *p* = .015). The other groups were not significantly different from each other (*ps* > .05). Fig 5 shows those results graphically. We also conducted the ANCOVA using the entire TA group, and the results showed the significant main effect of mathematics achievement groups in LMA, *F*(3, 211) = 3.10, *p* < .05, ƞ_p_^2^ = .04, 1 - β = .72.

## Discussion

Zan et al. have argued that “the most important problem for research on affect in mathematics is the understanding of the interrelationship between affect and cognition” (p.117) [72]. The current study revealed that mathematical metacognition mediated the relationship between children’s MA and word problem solving (WPS), after controlling for IQ. Regarding the group differences in mathematical metacognition and MA, the LA children exhibited lower levels of LMA than the MLD children with IQ partialled out. Moreover, the MLD children exhibited deficits in self-image, and LMA, but not MEA, compared to the TA and HA students.

Research in the domain of reading has revealed that test anxiety has a harmful effect on metacognitive word knowledge and influences performance in reading comprehension tasks [34]. Research in the mathematical domain has also found that individuals with higher anxiety benefit from having higher levels of metacognition when performing mathematical tasks [35]. Our results showed MA to be negatively related to the mathematical metacognition of 10-year-old children and subsequently related to WPS performance. This finding provides insight into factors that may mediate poor WPS performance which emerged under pressure in mathematics. This mediation effect also suggests that metacognition can counter the negative or stressful perceptions in mathematical performance.

It is worthy to note that the relationship among mathematics anxiety, metacognition, and word problem solving is complicated. First, in the longitudinal view, prior mathematical performance may be related to later mathematics anxiety [36]. However, there is also the possibility that mathematics anxiety may exist when children begin to learn mathematics in a formal academic setting [56]. In the present study, the mathematics anxiety measured here was a general fear or tension associated with anxiety-provoking situations that involve interaction with math in a wide variety of ordinary situations. The development of such anxiety may be related more globally to poor math performance instead of only word problem solving performance. It is here proposed that testing the “WPS-> MA-> metacognition” or “WPS-> metacognition-> MA” or “metacognition-> WPS—> MA” direction path models may be more valuable in longitudinal studies.

Second, the relationship “MA-> WPS-> metacognition” may also exist. Mathematics anxiety may be negatively related to WPS and may impact children’s mathematical metacognition. However, the mathematical metacognition we measured was a self-assessment on general metacognition about mathematical learning, and we measured it before WPS. Both MA and metacognition may have deep developmental origins (perhaps construed as trait variables) and short term origins (perhaps construed as state variables). Note that testing word problem solving may have only short-term effects, and the prediction that WPS would lead to metacognition could not be explained.

Third, even though the arrows in Kulm’s model mentioned in the introduction started from attitude, we acknowledged that attitudes are important both as independent and dependent variables. Similarly, metacognition might predict mathematics anxiety, and it might be related to WPS. However, only a few previous works have reviewed metacognition as it is related specifically to mathematics anxiety, and research about these three variables is sparse.

Jain and Dowson used structural equation modeling and found self-efficacy to be a mediating variable between self-regulation and mathematics anxiety [31]. That study was cross-sectional, although the aim was to find causal ordering. Consequently, the conclusion should be interpreted cautiously. As previously mentioned, Legg and Locker found that metacognition appeared to reduce the impact of anxiety on performance [35]. This work did not offer a holistic statistical analysis of the three variables. Another cross-cultural study [73] also showed a relationship between test anxiety and mathematical self-concept. Participants from Korea and Japan demonstrated low mathematical self-concept and high mathematics anxiety despite their high mathematical performance scores.

Past research includes investigations of the relationship between metacognition and anxiety, but existing studies have come to conflicting conclusions regarding the relationship among the three variables evaluated here. The purpose of the present study was to offer information regarding the role of metacognition in the relationship of MA and WPS. Considering the possibility of prediction from metacognition to anxiety, we ran the path models of “metacognition-> MA-> WPS” (see S1 Table, S1 Fig and S2 Fig), but the results did not show any mediating effect of MA on the relationship between metacognition and WPS.

In addition, many investigations have focused on cognitive deficits in children with MLD and their LA peers (e.g. [74, 75]), but there has been little or if any progress toward elaborating emotional functioning in these two groups [44]. Comparisons between MLD and LA students in the present study showed some intriguing differences. The children with MLD showed higher LMA and lower self-image than did those in the LA group. Because the difference in self-image between MLD and LA group was almost significant, caution should be used when generalizing the result. These results indicated that these two groups should not be conflated [42]. The present findings also suggest that one benefit of mathematical metacognition may be related to promoting beliefs about, and feelings of self-efficacy with respect to, the MLD children’s mathematical abilities. Moreover, helping MLD children reduce anxiety during the mathematics learning process should be incorporated into future interventions.

Results in the children with MLD also showed that MA and metacognition related to mathematical performance. They exhibited lower self-image, but higher levels of LMA, than did their TA and HA peers. Children classified with mathematical learning difficulty at some point experienced considerable failure and negative competence feedback at school. These experiences would likely be internalized and represented in a more negative view of self [76]. Our results showed self-image to be a powerful variable related to children’s mathematics performance. Self-image may reflect a wide range of related variables. Some studies have suggested that self-image is related to extended effort and persistence [77]. Individuals with higher mathematical self-image may interact with their teachers more frequently and may spend more time on tasks than students with lower self-concepts [38]. Because these variables behind self-image, such as interaction with teachers, may also be related to children’s performance, further research is needed to control these variables and explore the relationship between self-image and mathematical performance.

In the present study, no group differences were observed with respect to self-regulation, strategies, or motivation. We did not measure children’s strategies and self-regulation when they were doing mathematics tasks. Instead, we measured their general mathematical metacognitive strategies and self-regulation ability. For example, children had to evaluate situations in which “after I finish mathematics assignments, I review the main points in order to make sure I did learn the new knowledge.” Such strategies represented the basic strategies children used when facing similar supposed situations. It is possible that the basic strategies in children with MLD were sufficient, but when they were performing real mathematical tasks, the tasks may require more detailed and flexible strategies to monitor, adjust, and reflect upon the problem-solving process. These specific strategies may show differences among four mathematics achievement groups. The results suggested that determining the specificity of the metacognitive strategies used (general/specific) may be a useful way to identify children with MLD in the future.

Meanwhile, in the present study, children’s motivation to engage in mathematical learning reflected the impacts of their parents and teachers. For example, children were asked to rate the statement “my parents asked me to learn mathematics thoroughly.” Chinese parents and teachers tend to push children hard [78, 79], and this might be one of the reasons why the levels of motivation in the four achievement groups showed little difference. However, more research is needed.

Higher LMA in the MLD group indicates that the students might worry about their mathematics learning processes. LMA is a type of dynamic anxiety that involves children applying cognitive resources to ruminating on anxious thoughts and thus limiting the cognitive capacities available to organize WPS strategies. The data also point to the conclusion that MLD children might feel more nervous and anxious due to the process of learning mathematics rather than due to other people’s evaluations of them. Both the surroundings that are rich in mathematical information and mathematics problem solving settings may increase their anxious thoughts and then lower their performance. Indeed, recent research [80] has begun to focus on the early signs of LMA in young children in kindergarten, when these children view pictures of natural mathematics information in daily life and in situations involving simple mathematics. All of these findings are informative for schools in terms of the means by which to respond to the learning challenges of children, particularly children with MLD.

The results of the present study suggest a variety of avenues for potentially productive research, with additional possible practical applications. The present study used Chinese students. A few studies have shown that Confucian Asian students experience higher levels of mathematics anxiety and mathematics self-doubt than do students from other parts of the world [73, 81]. Because mathematics is a major key to success in so many fields, it is therefore easily conceivable that those affective elements impact mathematics learning, and possibly subsequent achievement in areas which rely on mathematics skills (e.g. traditional sciences, economics, and accounting). Even though the present study did not specifically test for cultural differences, there is a possibility that behavioral and psychosocial outcomes may differ across cultures, with special emphasis on Chinese vs. western culture. This possibility will need to be investigated in the future.

Another limitation warrant cautious consideration of these results. The present study was a cross-sectional study, and we did not experimentally manipulate anxiety and metacognition. It is important to recognize that the use of structural equation modeling does not automatically warrant creditability to any knowledge claim on a causal relationship. Longitudinal data would enable an estimation of the causal effects of metacognition and mathematics anxiety on word problem solving taking into account previous levels of each of these variables. We suggest that future studies could incorporate more diverse samples, providing longitudinal data, in order to verify the results of the study under more robust sampling and statistical conditions.

We also emphasize that this study only examined the structural relationship between metacognition, operationalized using a particular scale, and a particular (albeit important) form of mathematics performance, WPS. Further studies should record specific metacognitive behaviors, such as the types of strategies employed, during the solving of word problems to extend these results [82]. Other types of mathematics problems remain to be explored.

In that same vein, many of the correlations with WPS and with mathematics achievement scores are relatively small (see Table 1), none exceeding .28 (about 8% variance accounted for). While significant, those values strongly suggest that additional variables—likely including cognitive, metacognitive, and affective elements—play important roles in mathematics learning and performance. Those additional variables remain to be identified.

In the present study, working memory was not tested and the study only controlled for children’s intelligence score, because general intelligence is a critical control variable used for screening children with MLD, and because general intelligence here believed to represent individuals’ ability to process information. General intelligence indicates a variety of cognitive variables (e.g. reasoning ability, comprehension), and many researchers have suggested that general intelligence and working memory may share common variance [83–85]. Future studies that control for working memory or investigate the relationship among working memory, metacognition, mathematical anxiety, and word problem solving are planned. Besides, we acknowledge that reading ability is likely to be a covariate in predicting the degree of success with word problems, beyond general intelligence. While we did account for verbal IQ, reading ability was not assessed directly. Although pictorial illustrations were supplied for each word problem to help students interpret the text, future research should either include a control group without pictorial illustrations or directly measure reading ability. Those approaches were not feasible in the present study due to constraints on access to the children.

Additionally, the scales we used to measure metacognition and mathematics anxiety are only two possible ways to evaluate the relevant variables. Other convergent measures clearly would be highly desirable. Moreover, the measures we used are ordinal, and the range of ratings available is typically limited, even though such measures are widely used in behavioral studies. Future research should attempt to explore the fine gradations of the underlying variable that are impossible to discern with the measures we employed.

We tested fourth-grade children, all around 10 years of age and found affective and metacognitive correlates of mathematics performance. Mathematics education in China begins before that age and continues through all educational levels. It seems reasonable to propose that the patterns of affective and metacognitive influences seen across school ages might vary. It would therefore be advantageous to evaluate such patterns, and developmental changes in them, in students both younger and older than those we tested.

Finally, the small number of MLD students calls for caution in interpreting the path diagram of the three variables to examine the mediating effects in MLD children. Future research will need to test those relationships with larger samples of MLD children.

## Summary

This study examined effects of two important affective and cognitive variables (i.e. mathematical anxiety and mathematical metacognition) on children’s word problem solving abilities and explored the differences between four mathematics achievement groups. The results help identify the critical roles of, and relationships between, the two categories of variables in relation to children’s mathematical learning. Application of these outcomes has the potential to positively influence the formulation of targeted education and intervention plans for different groups. The present study also provides theoretical support for teachers seeking to decrease the effect of student mathematics anxiety on WPS from the new perspective of metacognitive intervention training.

## Supporting Information

S1 Fig Model 3: Partially mediated model with MA as a mediator and IQ partialled out. LMA = learning mathematics anxiety, MEA = mathematical evaluation anxiety. WPS = word problem solving. *** *p* < .001, ** *p* < .01.
(TIF)

S2 Fig Model 4: Fully mediated model with MA as a mediator and IQ partialled out. LMA = learning mathematics anxiety, MEA = mathematical evaluation anxiety. WPS = word problem solving. *** *p* < .001, ** *p* < .01.
(TIF)

S1 Table Fit indices for the structural equation modeling of mathematical metacognition, mathematics anxiety, and word problem solving with MA as a mediator. (DOC)

## References

[1] Mayer RE, Wittrock MC. Problem solving In: Alexander PA, Winne PH, editors. Handbook of educational psychology (2nd ed). Mahwah, New Jersey: Erlbaum; 2006 pp. 287–303.

[2] Sajadi M, Amiripour P, Rostamy-Malkhalifeh M. The Examining mathematical word problems solving ability under efficient representation aspect. Mathematics Education Trends and Research. 2013; 2013: 1–11.

[3] Ahmad A, Tarmizi RA, Nawawi M. Visual representations in mathematical word problem solving among form four students in Malacca. Procedia Soc Behav Sci. 2010; 8: 356–361.

[4] Montague M. Student perception, mathematical problem solving, and learning disabilities. Remedial Spec Educ. 1997;18: 46–53.

[5] Furinghetti F, Morselli F. Every unsuccessful problem solver is unsuccessful in his or her own way: affective and cognitive factors in proving. Educational Studies in Mathematics. 2009;70: 71–90.

[6] Hoffman B. “I think I can, but I'm afraid to try”: The role of self-efficacy beliefs and mathematics anxiety in mathematics problem-solving efficiency. Learn Individ Differ. 2010;20: 276–283.

[7] Hannula MS. Affect in mathematics education In: Lerman S, editor. Encyclopedia of Mathematics Education. Dordrecht: Springer; 2014 pp. 23–27.

[8] Richardson FC, Suinn RM. The Mathematics Anxiety Rating Scale: Psychometric data. J Couns Psychol. 1972; 19: 551.

[9] Karimi A, Venkatesan S. Mathematics anxiety, mathematics performance and academic hardiness in high school students. International Journal of Educational Sciences. 2009;1: 33–37.

[10] Chiu LH, Henry LL. Development and validation of the Mathematics Anxiety Scale for Children. Meas Eval Couns Dev. 1990;23: 121–127.

[11] Guven B, Cabakcor BO. Factors influencing mathematical problem-solving achievement of seventh grade Turkish students. Learn Individ Differ. 2013;23: 131–137.

[12] Alikamar MA, Alamolhodaei H, Radmehr F. The role of Metacognition on effect of Working Memory Capacity on students' mathematical problem solving. European Journal of Child development, Education and Psychopathology. 2013;1: 125–139.

[13] Johnson ES, Humphrey M, Mellard DF, Woods K, Swanson HL. Cognitive processing deficits and students with specific learning disabilities: A selective meta-analysis of the literature. Learn Disabil Q. 2010;33: 3–18.

[14] Zheng X, Swanson HL, Marcoulides GA. Working memory components as predictors of children’s mathematical word problem solving. J Exp Child Psychol. 2011;110: 481–498. 10.1016/j.jecp.2011.06.001 21782198

[15] Swanson HL. Influence of metacognitive knowledge and aptitude on problem solving. Journal of Educational Psychology; J Educ Psychol. 1990;82: 306.

[16] Veenman MV, Spaans MA. Relation between intellectual and metacognitive skills: Age and task differences. Learn Individ Differ. 2005;15: 159–176.

[17] Rosenzweig C, Krawec J, Montague M. Metacognitive strategy use of eighth-grade students with and without learning disabilities during mathematical problem solving: A think-aloud analysis. J Learn Disabil. 2011;44: 508–520. 10.1177/0022219410378445 21971084

[18] Flavell JH. Metacognitive aspects of problem solving. The nature of intelligence. 1976;12: 231–235.

[19] Panaoura A, Philippou G. The developmental change of young pupils' metacognitive ability in mathematics in relation to their cognitive abilities. Cogn Dev. 2007;22: 149–164.

[20] Panaoura A, Philippou G. The Construct Validity of an Inventory for the Measurement of Young Pupils' Metacognitive Abilities in Mathematics. International Group for the Psychology of Mathematics Education. 2003;3: 437–444.

[21] Jacobse AE, Harskamp EG. Towards efficient measurement of metacognition in mathematical problem solving. Metacogn Learn. 2012;7: 1–17.

[22] Cornoldi DLC. Mathematics and metacognition: What is the nature of the relationship? Mathematical cognition. 1997;3: 121–139.

[23] Montague M. Self-regulation strategies to improve mathematical problem solving for students with learning disabilities. Learn Disabil Q. 2008;31: 37–44.

[24] van der Stel M, Veenman MV. Relation between intellectual ability and metacognitive skillfulness as predictors of learning performance of young students performing tasks in different domains. Learn Individ Differ. 2008;18: 128–134.

[25] Teong SK. The effect of metacognitive training on mathematical word-problem solving. Journal of Computer Assisted Learning. 2003;19: 46–55.

[26] Pennequin V, Sorel O, Mainguy M. Metacognition, executive functions and aging: The effect of training in the use of metacognitive skills to solve mathematical word problems. J Adult Dev.2010;17: 168–176.

[27] Ashcraft MH, Kirk EP. The relationships among working memory, math anxiety, and performance. J Exp Psychol Gen. 2001;130: 224 11409101 10.1037//0096-3445.130.2.224

[28] Ashcraft MH. Math anxiety: Personal, educational, and cognitive consequences. Curr Dir Psychol Sci. 2002;11: 181–185.

[29] Ashcraft MH, Moore AM. Mathematics anxiety and the affective drop in performance. J Psychoeduc Assess. 2009;27: 197–205.

[30] Kesici Ş, Baloğlu M, Deniz M. Self-regulated learning strategies in relation with statistics anxiety. Learn Individ Differ. 2011;21: 472–477.

[31] Jain S, Dowson M. Mathematics anxiety as a function of multidimensional self-regulation and self-efficacy. Contemp Educ Psychol. 2009;34: 240–249.

[32] Ahmed W, Minnaert A, van der Werf G, Kuyper H. Perceived social support and early adolescents' achievement: The mediational roles of motivational beliefs and emotions. J Youth Adolesc. 2010;39: 36–46. 10.1007/s10964-008-9367-7 20091215 PMC2796962

[33] Bandalos DL, Yates K, Thorndike-Christ T. Effects of math self-concept, perceived self-efficacy, and attributions for failure and success on test anxiety. J Educ Psychol. 1995;87: 611.

[34] Everson HT, Smodlaka I, Tobias S. Exploring the relationship of test anxiety and metacognition on reading test performance: A cognitive analysis. Anxiety Stress Coping. 1994;7: 85–96.

[35] Legg AM, Locker L Jr. Math performance and its relationship to math anxiety and metacognition. N AM J Psychol. 2009;11: 471–486.

[36] Ma X, Xu J. The causal ordering of mathematics anxiety and mathematics achievement: a longitudinal panel analysis. J Adolesc. 2004;27: 165–179. 15023516 10.1016/j.adolescence.2003.11.003

[37] Jansen BR, Louwerse J, Straatemeier M, Van der Ven SH, Klinkenberg S, Van der Maas HL. The influence of experiencing success in math on math anxiety, perceived math competence, and math performance. Learn Individ Differ. 2013;24: 190–197.

[38] Kulm G. Research on mathematics attitude In: Shumway RJ, editors. Research in mathematics education. Reston: National Council of Teachers of Mathematics; 1980 p.380.

[39] Reyes LH. Affective variables and mathematics education. Elem Sch J. 1984;84: 558–581.

[40] American Psychiatric Association. Diagnostic and statistical manual of mental disorders, text revision (DSM-IV-TR). Washington, DC: American Psychiatric Association; 2000.

[41] Swanson HL, Harris KR, Graham S. Handbook of learning disabilities. New York: Guilford Press; 2013.

[42] Geary DC, Hoard MK, Byrd Craven J, Nugent L, Numtee C. Cognitive mechanisms underlying achievement deficits in children with mathematical learning disability. Child Dev. 2007;78: 1343–1359. 17650142 10.1111/j.1467-8624.2007.01069.x PMC4439199

[43] Murphy MM, Mazzocco MMM, Hanich LB, Early MC. Cognitive characteristics of children with mathematics learning disability (MLD) vary as a function of the cutoff criterion used to define MLD. J Learn Disabil. 2007;40: 458–478. 17915500 10.1177/00222194070400050901

[44] Geary DC. Consequences, characteristics, and causes of mathematical learning disabilities and persistent low achievement in mathematics. J Dev Behav Pediatr. 2011;32: 250 10.1097/DBP.0b013e318209edef 21285895 PMC3131082

[45] Bryant DP, Bryant BR, Hammill DD. Characteristic behaviors of students with LD who have teacher-identified math weaknesses. J Learn Disabil. 2000;33: 168–177. 15505946 10.1177/002221940003300205

[46] González JEJ, Espinel AIG. Strategy choice in solving arithmetic word problems: Are there differences between students with learning disabilities, GV poor performance and typical achievement students? Learn Disabil Q. 2002;25: 113–122.

[47] Montague M, Applegate B. Middle school students' mathematical problem solving: An analysis of think-aloud protocols. Learn Disabil Q. 1993;16: 19–32.

[48] Garrett AJ, Mazzocco MM, Baker L. Development of the metacognitive skills of prediction and evaluation in children with or without math disability. Learn Disabil Res Pract. 2006;21: 77–88. 20084181 10.1111/j.1540-5826.2006.00208.x PMC2806675

[49] Stone CA, May AL. The accuracy of academic self-evaluations in adolescents with learning disabilities. J Learn Disabil. 2002;35: 370–383. 15493246 10.1177/00222194020350040801

[50] Desoete A, Roeyers H, Buysse A. Metacognition and mathematical problem solving in grade 3. J Learn Disabil. 2001;34: 435–447. 15503592 10.1177/002221940103400505

[51] Desoete A, Roeyers H, De Clercq A. Children with mathematics learning disabilities in Belgium. J Learn Disabil. 2004;37: 50–61. 15493467 10.1177/00222194040370010601

[52] Fuchs LS, Fuchs D, Prentice K. Responsiveness to Mathematical Problem-Solving Instruction. J Learn Disabil. 2004;37: 293–306. 15493402 10.1177/00222194040370040201

[53] Landerl K, Bevan A, Butterworth B. Developmental dyscalculia and basic numerical capacities: A study of 8–9-year-old students. Cognition. 2004;93: 99–125. 15147931 10.1016/j.cognition.2003.11.004

[54] Nelson JM, Harwood H. Learning Disabilities and Anxiety: A Meta-Analysis. J Learn Disabil. 2011;44: 3–17. 10.1177/0022219409359939 20375288

[55] Bryan JH, Sonnefeld LJ, Grabowski B. The Relationship between Fear of Failure and Learning Disabilities. Learn Disabil Q. 1983;6: 217–222.

[56] Wu SS, Barth M, Amin H, Malcarne V, Menon V. Math anxiety in second and third graders and its relation to mathematics achievement. Front Psychol. 2012;3: 1–11. 10.3389/fpsyg.2012.00001 22701105 PMC3369194

[57] Rousselle L, Noël MP. Basic numerical skills in children with mathematics learning disabilities: A comparison of symbolic vs non-symbolic number magnitude processing. Cognition. 2007;102: 361–395. 16488405 10.1016/j.cognition.2006.01.005

[58] Geary DC. Learning disabilities in arithmetic: Problem-solving differences and cognitive deficits In Swanson HL, Harris KR, Graham S, editors. Handbook of learning disabilities. New York: The Guilford Press; 2003 pp. 199–212.

[59] Gold AB, Ewing-Cobbs L, Cirino P, Fuchs LS, Stuebing KK, Fletcher JM. Cognitive and behavioral attention in children with math difficulties. Child Neuropsychol. 2012;19: 420–437. 10.1080/09297049.2012.690371 22686370 PMC4155495

[60] Desoete A, Ceulemans A, De Weerdt F, Pieters S. Can we predict mathematical learning disabilities from symbolic and non-symbolic comparison tasks in kindergarten? Findings from a longitudinal study. Br J Educ Psychol. 2012;82: 64–81. 10.1348/2044-8279.002002 21199482

[61] Mazzocco MM, Myers GF, Lewis KE, Hanich LB, Murphy MM. Limited knowledge of fraction representations differentiates middle school students with mathematics learning disability (dyscalculia) versus low mathematics achievement. J Exp Child Psychol. 2013;115: 371–387. 10.1016/j.jecp.2013.01.005 23587941 PMC4000738

[62] Von Aster MG, Shalev RS. Number development and developmental dyscalculia. Dev Med Child Neurol. 2007;49: 868–873. 17979867 10.1111/j.1469-8749.2007.00868.x

[63] Mazzocco MM, Devlin KT. Parts and ‘holes': Gaps in rational number sense among children with vs. without mathematical learning disabilities. Dev Sci. 2008;11: 681–691. 10.1111/j.1467-7687.2008.00717.x 18801123

[64] Mazzocco MM, Feigenson L, Halberda J. Impaired acuity of the approximate number system underlies mathematical learning disability (dyscalculia). Child Dev. 2011;82: 1224–1237. 10.1111/j.1467-8624.2011.01608.x 21679173 PMC4411632

[65] Hoard MK, Geary DC, Byrd-Craven J, Nugent L. Mathematical cognition in intellectually precocious first graders. Dev Neuropsychol. 2008;33: 251–276. 10.1080/87565640801982338 18473199

[66] Zhang HC, Wang XP. Standardization research on Raven’s Standard Progressive Matrices in China. Acta Psychologica Sinica. 1989;2: 113–121.

[67] Zhang HC. The revision of WISC-IV Chinese version. Psychological Science (China). 2009;32: 1177–1179.

[68] Liu J, Sun XT. Chinese mathematics curriculum standards of Full-time compulsory education reading. Beijing: Beijing Normal University Publishing Group; 2002.

[69] Hao JJ, Qi L, Chen YH. The Metacognitive Ability of Sixth-Year Primary School Students with Mathematics Learning Disabilities and Their Performance on Application Problem Tests. Chinese Journal of Special Education. 2011;128: 52–57.

[70] Plake BS, Parker CS. The development and validation of a revised version of the Mathematics Anxiety Rating Scale. Educ Psychol Meas. 1982;42: 551–557.

[71] Lai YH, Zhu XS, Huang DQ, Chen YH. A Comparison between Children with Mathematics Learning Difficulties and Children with Normal Mathematics Learning Abilities in Spatial Abilities in 3th to 6th Grades. Studies of Psychology and Behavior. 2014;12: 36–44.

[72] Zan R, Brown L, Evans J, Hannula MS. Affect in mathematics education: An introduction. Educ Stud Math. 2006;63: 113–121.

[73] Lee J. Universals and specifics of math self-concept, math self-efficacy, and math anxiety across 41 PISA 2003 participating countries. Learn Individ Differ. 2009;19: 355–365.

[74] Geary DC, Hoard MK, Bailey DH. Fact retrieval deficits in low achieving children and children with mathematical learning disability. J Learn Disabil. 2011. 10.1177/0022219410392046 PMC3163113 21252374

[75] Geary DC, Hoard MK, Nugent L, Bailey DH. Mathematical cognition deficits in children with learning disabilities and persistent low achievement: A five-year prospective study. J Educ Psychol. 2012;104: 206. 27158154 10.1037/a0025398 PMC4855881

[76] Grolnick WS, Ryan RM. Self-perceptions, motivation, and adjustment in children with learning disabilities: A multiple group comparison study. J Learn Disabil. 1990;23: 177–184. 2313191 10.1177/002221949002300308

[77] Hoffman B, Spatariu A. The influence of self-efficacy and metacognitive prompting on math problem-solving efficiency. Contemp Educ Psychol. 2008;33: 875–893.

[78] Lu HD. Focus on learning stress of Chinese children: The puzzledom and the way out. Journal of Northeast Normal University (Philosophy and Social Sciences). 2008;236: 24–28.

[79] Long AB, Fan W, Jin XH. Measurement and attribution model construction on academic stress of primary and secondary school students. Journal of Educational Studies. 2013;9: 121–128.

[80] Aarnos E, Perkkilä P. Early Signs of Mathematics Anxiety? Procedia Soc Behav Sci. 2012;46: 1495–1499.

[81] van de Vijver FJR. On the elusive nature of high Chinese achievement. Learn Individ Differ. 2010;20: 574–576.

[82] Ostad SA, Sorensen PM. Private speech and strategy-use patterns bidirectional comparisons of children with and without mathematical difficulties in a developmental perspective. J Learn Disabil. 2007;40: 2–14. 17274544 10.1177/00222194070400010101

[83] Jaeggi SM, Buschkuehl M, Jonides J, Perrig WJ. Improving fluid intelligence with training on working memory. Proc Natl Acad Sci U S A. 2008;105: 6829–6833. 10.1073/pnas.0801268105 18443283 PMC2383929

[84] Ackerman PL, Beier ME, Boyle MO. Working memory and intelligence: The same or different constructs? Psychol Bull. 2005;131: 30 15631550 10.1037/0033-2909.131.1.30

[85] Conway AR, Kane MJ, Engle RW. Working memory capacity and its relation to general intelligence. Trends Cogn Sci. 2003;7: 547–552. 14643371 10.1016/j.tics.2003.10.005

