# Supplementary figures and images for: Effects of Mathematics Anxiety and Mathematical Metacognition on Word Problem Solving in Children with and without Mathematical Learning Difficulties
Source: PLoS One. 2015 Jun 19;10(6):e0130570. doi: 10.1371/journal.pone.0130570 (PMC4474805; doi:10.1371/journal.pone.0130570)

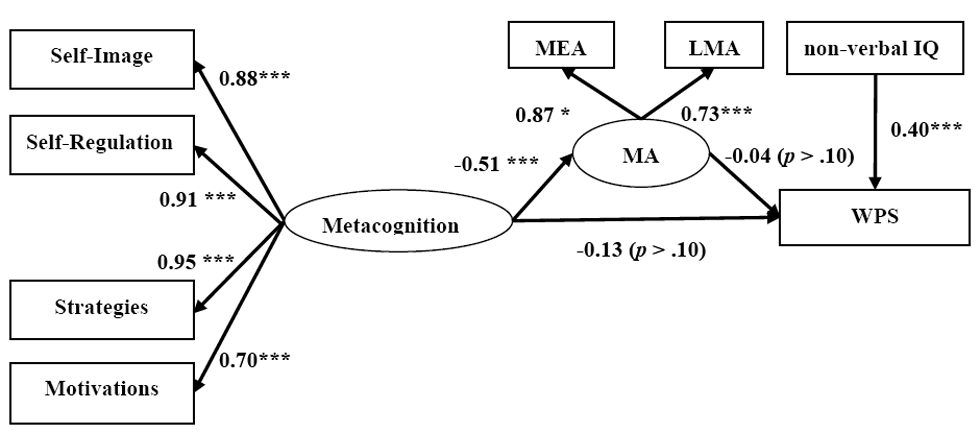

Supplement: S1 Fig — LMA = learning mathematics anxiety, MEA = mathematical evaluation anxiety. WPS = word problem solving. *** p < .001, ** p < .01. (TIF) [file pone.0130570.s001.tif]

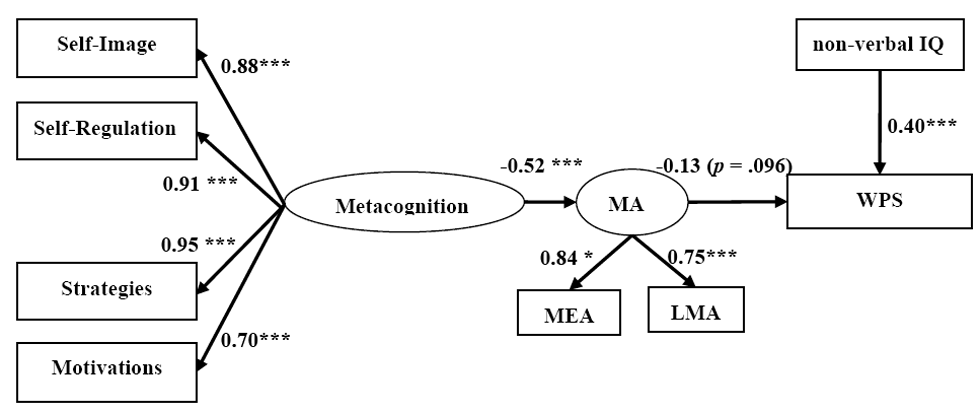

Supplement: S2 Fig — LMA = learning mathematics anxiety, MEA = mathematical evaluation anxiety. WPS = word problem solving. *** p < .001, ** p < .01. (TIF) [file pone.0130570.s002.tif]
