# Supplementary material for: Effects of Mathematics Anxiety and Mathematical Metacognition on Word Problem Solving in Children with and without Mathematical Learning Difficulties
Source: PLoS One. 2015 Jun 19;10(6):e0130570. doi: 10.1371/journal.pone.0130570 (PMC4474805; doi:10.1371/journal.pone.0130570)
Supplement: S1 Table — (DOC) [file pone.0130570.s003.doc]

**Table S1**

| ***Model*** | ***χ^2^*** | ***df*** | ***χ*^2^/*df*** | ***RMSEA*** | ***TLI*** | ***SRMR*** | ***CFI*** |
| --- | --- | --- | --- | --- | --- | --- | --- |
| **M3** | 37.54 | 18 | 2.09 | 0.070 | 0.97 | 0.05 | 0.98 |
| **M4** | 40.35 | 19 | 2.12 | 0.071 | 0.97 | 0.06 | 0.98 |

Note. Model 3: Partially mediated model with MA as a mediator. Model 4: Fully mediated model with MA as a mediator.
